# High-Accuracy Calibration Based on Linearity Adjustment for Eddy Current Displacement Sensor

**DOI:** 10.3390/s18092842

**Published:** 2018-08-28

**Authors:** Wei Liu, Bing Liang, Zhenyuan Jia, Di Feng, Xintong Jiang, Xiao Li, Mengde Zhou

**Affiliations:** Key Laboratory for Precision and Non-traditional Machining Technology of the Ministry of Education, Dalian University of Technology, Dalian 116024, China; liangbing2016@mail.dlut.edu.cn (B.L.); zy_jia@yeah.net (Z.J.); FD@mail.dlut.edu.cn (D.F.); 18390584678@163.com (X.J.); melixiao@mail.dlut.edu.cn (X.L.); mengdezl@163.com (M.Z.)

**Keywords:** eddy current displacement sensor, calibration, linearity adjustment, weighted support vector machine

## Abstract

High precision position control is essential in the process of parts manufacturing and assembling, where eddy current displacement sensors (ECDSs) are widely used owing to the advantages of non-contact sensing, compact volume, and resistance to harsh conditions. To solve the nonlinear characteristics of the sensors, a high-accuracy calibration method based on linearity adjustment is proposed for ECDSs in this paper, which markedly improves the calibration accuracy and then the measurement accuracy. After matching the displacement value and the output voltage of the sensors, firstly, the sensitivity is adjusted according to the specified output range. Then, the weighted support vector adjustment models with the optimal weight of the zero-scale, mid-scale and full-scale are established respectively to cyclically adjust the linearity of the output characteristic curve. Finally, the final linearity adjustment model is obtained, and both the calibration accuracy and precision are verified by the established calibration system. Experimental results show that the linearity of the output characteristic curve of ECDS adjusted by the calibration method reaches over 99.9%, increasing by 1.9–5.0% more than the one of the original. In addition, the measurement accuracy improves from 11–25 μm to 1–10 μm in the range of 6mm, which provides a reliable guarantee for high accuracy displacement measurement.

## 1. Introduction

Online precise displacement measurement and positioning are important parts of the manufacturing process quality inspection system [1]. Traditional methods are usually based on electronic theodolites, iGPS and laser trackers [2]. For electronic theodolites, the accuracy of angle measurement is up to 0.5"; however, the accuracy of distance measurement only reaches millimeter level due to the introduction of the sight distance method [3]. The precision of iGPS and laser trackers are very high in wide range measurements. However, due to the limitation of equipment response frequency and manual auxiliary operation, they are only suitable for low dynamic measurements [4]. Eddy current displacement sensors (ECDSs), due to the micron resolution and the high dynamic response up to 20 kHz even in harsh environments and their low cost [5,6,7,8], are widely used in measurement of displacement or movement in aviation, shipbuilding and military and many other fields [9,10,11]. Victor Farm-Guoo Tseng and Huikai Xie demonstrated resonant inductive coupling-based eddy current sensing as a promising piston position sensing mechanism for large vertical displacement micromirrors and presented a frequency detection method to simultaneously sense the piston position and tilt angle of the mirror plate of large vertical displacement micromirrors with nanometer resolution [12,13]. Moreover, in order to achieve closed-loop control of the inclined angle of the mirrors on the satellite communication equipment, Micro-Epsilon (Ortenburg, Germany) provided a high-accuracy ECDS system to accurately measure the mirror rotation angle with angular resolutions better than one microrad [14]. Meanwhile, due to the miniaturized design of the ECDS and its advantages of the resistance to oil contamination and high temperature, it was also used in turbocharger test benches for the detection of turbocharger performance under different stress states [15].

The resolution of the ECDS can reach micron level or even below [16,17,18]. However, the relatively low sensitivity and linearity seriously restrict the detection accuracy because it is usually calibrated by a linear model before industrial use, neglecting the nonlinearity. In order to obtain better performance of the sensor, much research has been conducted for improving the sensitivity, optimizing the circuit design and high-accuracy calibration.

From the aspect of optimizing the circuit design, Zilian Qu explored the relationship between the measurement sensitivity and sensor parameters using an equivalent circuit model, and conducted experiments with a series of Cu films of different thickness, which showed that the sensor made of a lower resistant multi-wire Cu line had better sensitivity [19]. Mohammad Reza Nabavi discussed key design aspects and their interfaces for ECDSs, and employed second-order LC cross-coupled oscillators as the front-end stage to improve the precision during the measurements [20]. Darko Vyroubal and Igor Lacković analyzed the optimal operating frequency for minimum influence of temperature (20–600 °C) on displacement measurement to reduce the displacement measurement ambiguity [21]. Many other research works have also been conducted, such as using a small flat sensing coil for mechanical stability and compactness [22], increasing the excitation current to enhance detection sensitivity of amplitude parameter [23], etc. However, in addition to complex workload, the ECDSs still suffer from a certain degree of nonlinearity due to the output characteristics of the designed circuit. Thus, subsequent high-accuracy calibration of ECDS is necessary. Xing Ding proposed a piecewise linear fitting method that increased the linearity to above 99%, while the accuracy reached within 30 μm in the range of 6 mm [24]. Hesheng Zhang constructed the inverse model of the ECDS using the radial basis function (RBF) neural network to achieve the high-precision calibration with an error less than 0.7%, which satisfied the requirement of the maglev system control [25]. Clough David proposed an in situ calibration method, which used the short range positional accuracy of the machine as a reference, employed least squares best fit of a low order polynomial, and verified the accuracy in a practical example [26]. Anish Babu adopted the sine function to record the relationship of every position and its output value within 3 mm range, and the calibration error of the sensor reached less than 0.6% [27,28]. However, whether the linear methods ignore the nonlinearity problem, or the nonlinear methods cause an overfitting problem in the calibration process will restrict the improvement of the accuracy.

In this paper, a high accuracy calibration method based on linearity adjustment has been proposed, which combines the weighted support vector machine and the cyclical linearity adjustments of the output characteristic curve, consist of mid-scale adjustments, zero-scale adjustments and full-scale adjustments. Meanwhile, both accuracy and precision of ECDs are also discussed. The rest of this paper is organized as follows: Section 2 briefly describes the displacement measurement principle of the ECDS, and the original sensitivity and linearity are tested. Section 3 details the proposed calibration method, which is based on the linearity adjustment models, and gives the final adjustment function. Section 4 verifies the calibration accuracy and precision, and conclusions are summarized in Section 5.

## 2. Original Output Characteristics Test

### 2.1. Relational Model of Displacement and Acquisition Voltage

**Figure 1:**
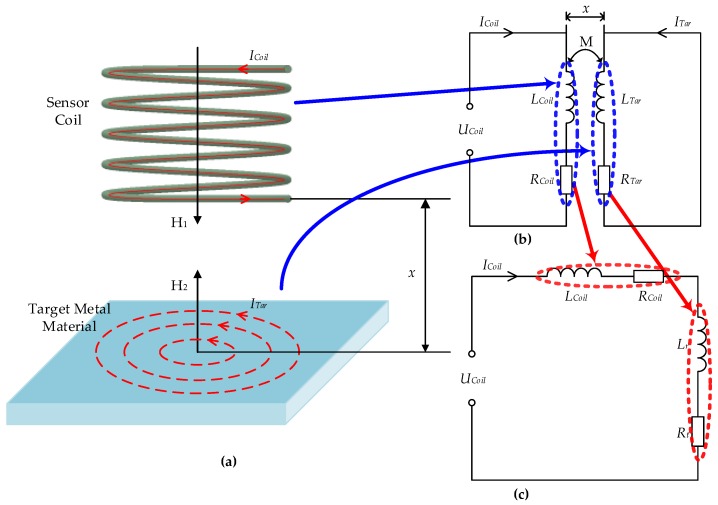
Principle of displacement measurement based on eddy current displacement sensor (ECDS): (**a**) principle model; (**b**) simplified circuit; (**c**) equivalent circuit.

The basic principle of ECDS to measure the displacement *x* of target metal materials is shown in Figure 1a. According to Faraday’s law, when an alternating current is applied to the sensor coil, an eddy current is generated in the target material, as shown in Figure 1b. The generated eddy current can be regarded as another induced coil, which has an influence on the voltage and current in the sensor coil at a different degree in accordance with *x*. Considering the induced coil as a reduced impedance, the equivalent circuit is shown in Figure 1c.

In Figure 1b,c, *U*, *I*, *R*, *L* denote the voltage, current, resistance and inductance, respectively. Subscript ‘*Coil*’, ‘*Tar*’, ‘*r*’ represent the sensor coil, target material, and the reduced impedance, respectively; *M* represents their inductance.

Then, the reduced impedance Δ*Z* can be:

(1) $$\Delta Z=\tilde{M}\left(R_{Tar}-j\omega L_{Tar}\right),$$

where $\tilde{M}=\omega^{2}M^{2}/\left[R_{Tar}^{2}+{\left(\omega L_{Tar}\right)}^{2}\right]$.

The following relationship exists between the current in the sensor coil and the one in target material:

(2) $${\dot{I}}_{Tar}={\dot{I}}_{Coil}\left[1-\frac{x/r}{\sqrt{{\left(x/r\right)}^{2}+1}}\right]\approx {\dot{I}}_{Coil}\left(1-\frac{x}{r}\right),$$

where *r* is the radius of the sensor coil. Meanwhile, ignoring the minor terms ${\left(x/r\right)}^{2}$ only when x/r≪1, the output curve of the sensor has a good linearity.

**Figure 2:**
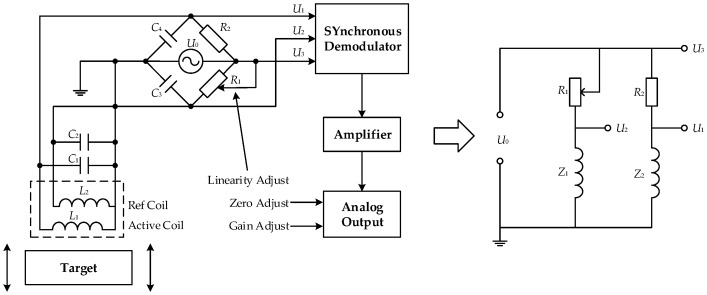
Measurement circuit and its simplified equivalent circuit.

In Figure 2a, according to Kirchhoff’s law, there is the following relationship:

(3) $$-j\omega M{\dot{I}}_{Coil}+\left(R_{Tar}+j\omega L_{Tar}\right){\dot{I}}_{Tar}=0.$$

Combining Equations (2) and (3), Equation (4) can be obtained:

(4) $$\tilde{M}=\frac{\omega^{2}M^{2}}{\left[R_{Tar}^{2}+{\left(\omega L_{Tar}\right)}^{2}\right]}={\left(1-\frac{x}{r}\right)}^{2}=1-2\frac{x}{r}+{\left(\frac{x}{r}\right)}^{2}.$$

Substituting Equation (4) into Equation (1) and ignoring the minor terms ${\left(x/r\right)}^{2}$, then Equation (5) can be obtained:

(5) $$\Delta Z=\left(1-2\frac{x}{r}\right)\left(R_{Tar}-j\omega L_{Tar}\right).$$

Equation (5) shows that the displacement values measured by the sensor is approximately linear to the reduced impedance of the target material.

In order to calculate the displacement change, a high-accuracy LC oscillation circuit needs to be established to convert the reduced impedance into voltage for subsequent acquisition. Measurement circuit is shown on the left side of Figure 2, and the simplified equivalent circuit is shown on the right.

The monitored voltage can be expressed as:

(6) $$\begin{array}{c} U_{2}=U_{0}-\frac{U_{0}}{R_{1}+Z_{1}}\cdot R_{1},\\ U_{1}=U_{0}-\frac{U_{0}}{R_{2}+Z_{2}}\cdot R_{2}, \end{array}$$

where the impedance of the reference coil $Z_{1}=1/\left(j\omega C_{3}+j\omega C_{2}-j{\left(\omega L_{2}\right)}^{-1}\right)$, and the impedance of the active coil $Z_{2}=1/\left(j\omega C_{4}+j\omega C_{1}-j{\left(\omega L_{1}\right)}^{-1}\right)$. The reference coil is set up to serve as the comparative value of the active coil and to facilitate subsequent adjustment of the voltage at the displacement x=0, which is completely shielded from the active coil and insensitive to displacement changes. The active coil is the sensing coil and its impedance changes as the displacement *x* changes.

Then, the voltage difference is recorded as $\Delta U=U_{2}-U_{1}$. When *x* changes, $Z_{2}$ changes to $Z_{2}^{\prime }=Z_{2}+\Delta Z_{2}\left(\Delta Z_{2}\ll Z_{2}\right)$. Thus, the variation of ΔU (ignoring the minor items) can be written as:

(7) $$d\left(\Delta U\right)=\Delta U^{\prime }-\Delta U=-\frac{U_{0}R_{2}}{{\left(R_{2}+Z_{2}\right)}^{2}}\Delta Z_{2}.$$

By Equation (7), we can get that the voltage difference d(ΔU) is linear to the reduced impedance $\Delta Z_{2}$. Meanwhile, combining the approximately linear relationship between the reduced impedance and the displacement, which is obtained by Equation (5), it can be concluded that the voltage difference d(ΔU) and the displacement *x* are linear.

### 2.2. Sensitivity and Linearity Test

**Figure 3:**
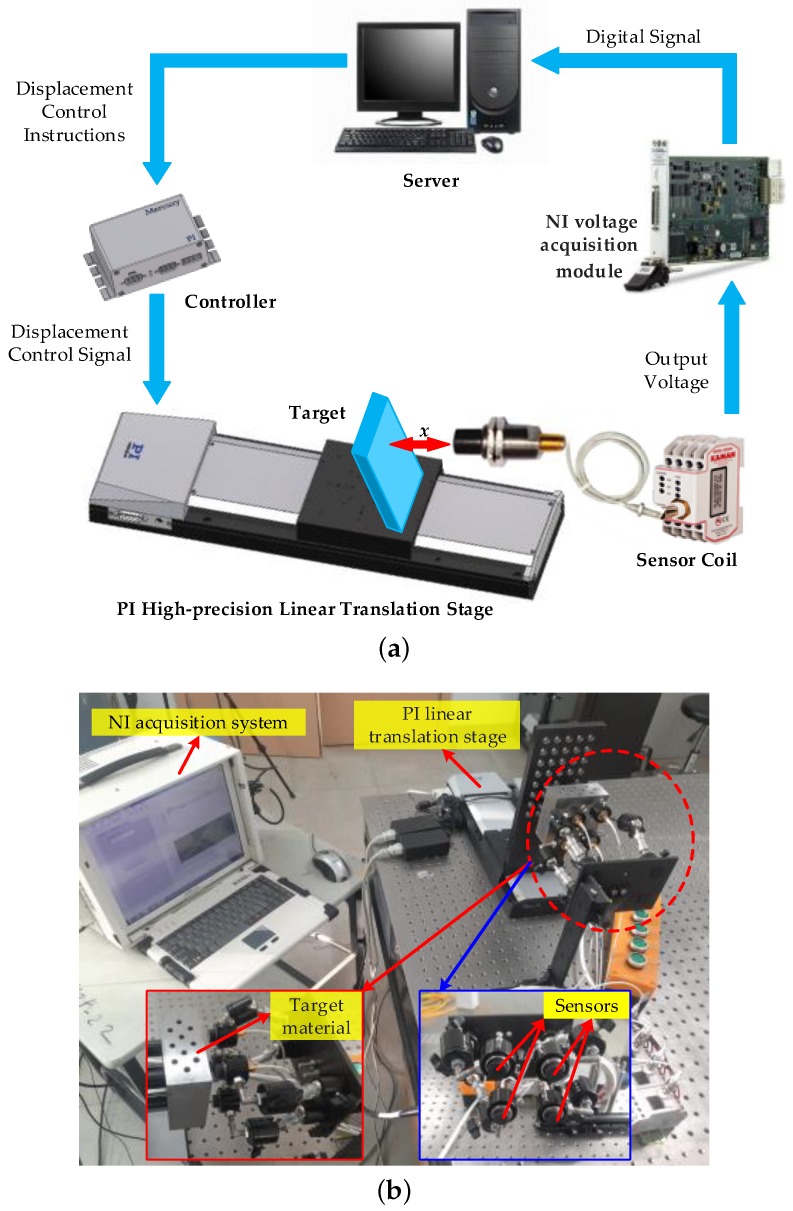
Calibration system flow and apparatus: (**a**) system flow; (**b**) calibration apparatus.

The original sensitivity and linearity are tested first in the process of displacement-voltage (x−u) calibration. Moreover, the system flow is shown in Figure 3a and the calibration apparatus is shown in Figure 3b, where a high-precision linear translation stage (PI, M-521.DD1) is employed for producing absolute displacement with the repeatability of 0.1 μm, and a voltage acquisition module (NI, PXI-6289) is employed to measure the sensor output voltage with the absolute accuracy of 2 mV.

**Figure 4:**
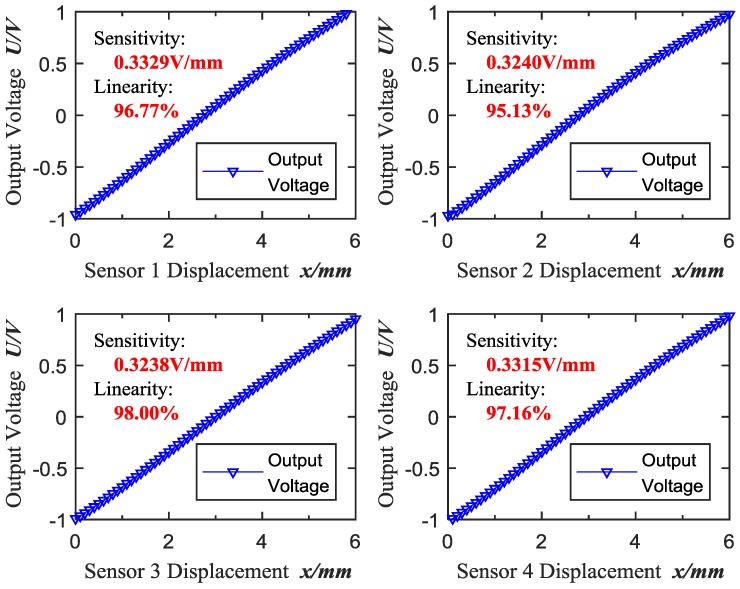
Original calibration data.

In the experiments, the displacement variation interval is 0.1 mm during the range of 0–6 mm, and the corresponding output voltages are collected at the same time. In this paper, four ECDSs (Kaman, KD2306-6U1, working temperature: 10–40 °C) are tested in order to avoid individual contingency. Consequently, the original calibration data is shown in Figure 4 and the original sensitivity and linearity of the tested ECDSs are calculated as shown in Table 1. In this paper, independent linearity [29] is adopted to evaluate the deviation of the ECDSs’ actual performance relative to the ideal characteristic curve (straight line, whose position is not limited, to minimize the deviation between the straight line and the actual performance).

As shown in Figure 4 and Table 1, the original output range is nearly at −1–+1 V with the low sensitivity around 0.33 V/mm and the poor linearity from 95% to 98%, which limits the accuracy of subsequent measurements. Thus, high-accuracy calibration of ECDs is significantly essential to improve further the accuracy for high precision process control of parts manufacturing and assembling.

## 3. Calibration Method Based on Linearity Adjustment Model

**Figure 5:**
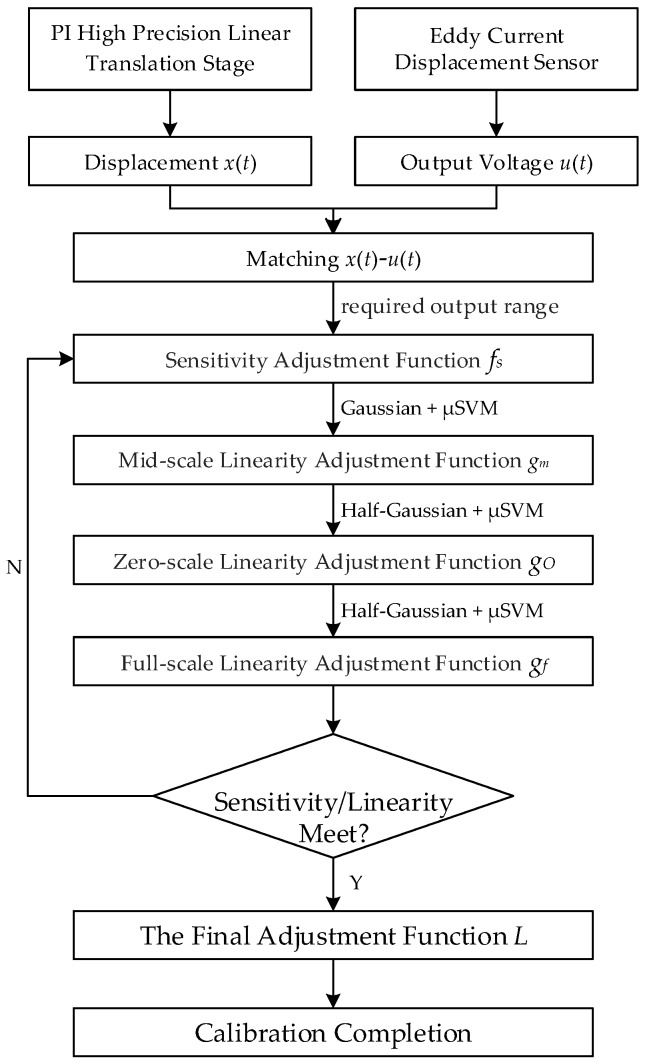
Calibration flow chart.

In order to calibrate the ECDs accurately and effectively, we propose a calibration method based on cyclic linearity adjustment, and the calibration flow is shown in Figure 5. The linearity adjustment model established in this paper is divided into the following steps:

(a) Firstly, adjust the sensitivity of the original calibration result by regulating the voltage *u* to the required output range;

(b) Then, partition adjustment is adopted on the account of targeted adjustments to the deviations of different regions; moreover, mid-scale, zero-scale and full-scale adjustments are determined for operation convenience and universality;

(c) Finally, the iterate adjustment function to perform overall fine-tuning until the sensitivity, linearity and required voltage output range meet the requirements.

### 3.1. Sensitivity Adjustment Function

The mapping relationship $f_{s}:u\to U$ between the actual sensor output value *u* and the required output value *U* is established so as to change and adjust the slope of the output characteristic curve (the sensitivity). The actual output value *u* and the required output value *U* satisfy:

(8) $$\left\{\begin{array}{c} u\in [u_{min},u_{max}],\\ U\in [U_{min},U_{max}]. \end{array}\right.$$

Then, the spatial linear mapping from *u* to *U* can be described by:

(9) $$f_{s}:u_{s}=U_{min}+\frac{u-u_{min}}{u_{max}-u_{min}}\left(U_{max}-U_{min}\right),$$

where $u_{s}$ is the output voltage after sensitivity adjustment.

According to the x−u calibration data of the sensors in Figure 4, it can be concluded that *u* satisfies u∈[−1 V, +1 V]. Then, the sensitivity can be maximized based on the input voltage range requirements of the acquisition module (NI, PXI-6289), which is limited to U∈[0 V, +10 V], and the sensitivity adjustment function can be described by:

(10) $$f_{s}:u_{s}=5\cdot \left(u+1\right).$$

**Figure 6:**
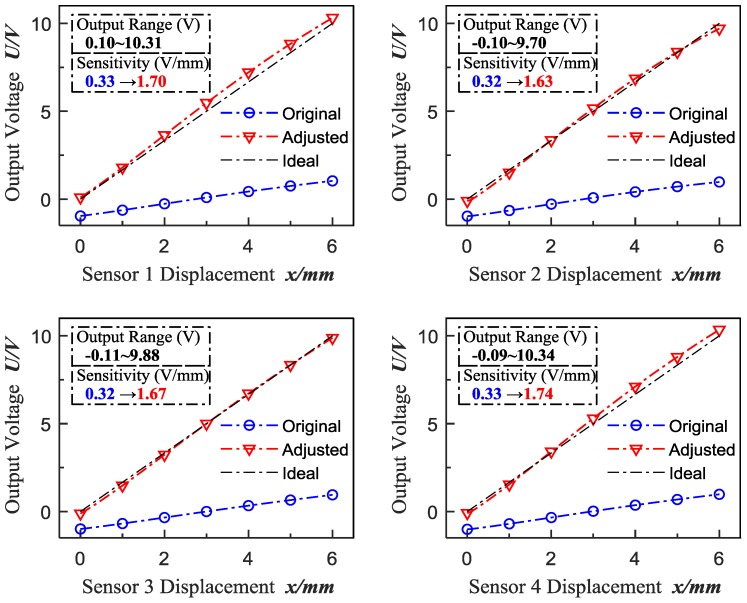
Curves of sensitivity adjustment results.

The sensitivity adjustment results are shown in Figure 6 and Table 2.

As shown in Figure 6 and Table 2, the adjusted output range is nearly at 0–+10 V with the sensitivity increased from around 0.33 V/mm to above 1.63 V/mm.

### 3.2. Mid-Scale Adjustment Function

After sensitivity adjustment by $f_{s}$, $u_{s}$ is obtained. Then, the mid-scale adjustment function based on weighted support vector machine (μSVM) will be established to adjust the linearity of $u_{s}$, which approaches *U*.

Weighted support vector machine is a commonly used machine learning method for solving classification or regression problems, which has a good performance in many practical problems [30,31]. In this paper, μSVM is adopted for two reasons: firstly, support vector machine (SVM) method can adjust the nonlinearity moderately avoiding overfitting, due to the introduction of slack variable; secondly, targeted nonlinearity adjustment is achieved by the determination of weight coefficient μ.

Based on the given training data set $D=\{\left(u_{si},U_{i}\right)|i=1,2,\cdots ,n\}$, $u_{si}\in R^{n}$, $U_{i}\in \left\{-1,+1\right\}$, the SVM method calculates the following optimization problem:

(11) $$\begin{array}{c} min\frac{1}{2}\omega_{T}\omega +C\sum_{i=1}^{n}\mu_{i}\xi_{i},\\ s.t.\;\left\{\begin{array}{c} U_{i}\left[\left(\omega \cdot u_{i}\right)+b\right]\geq 1-\xi_{i},\\ \xi_{i}\geq 0,i=1,2,\cdots ,n, \end{array}\right. \end{array}$$

where ω and *b* is the optimal hyper plane slope and intercept, respectively, *C* is the coefficient of the penalty function, $\xi_{i}$ is the slack variable and $\mu_{i}$ is the weight coefficient.

In order to make the measured values quickly and accurately tend to the ideal values, an optimal weight coefficient is established for the region to be adjusted. In this paper, Gauss function is selected to assign weights $\mu_{i}$ to the measured voltage values for its peak value and distribution range are easy to control, which is shown in Equation (12):

(12) $$\mu_{i}=\frac{1}{\sigma_{x}\sqrt{2\pi }}e^{-\frac{{\left(x-\bar{x}\right)}^{2}}{2\sigma_{x}^{2}}}.$$

In the process of mid-scale adjustment, the peak of adjustment amplitude is set at x=3. Thus, the average value $\bar{x}=3$ mm and standard deviation $\sigma_{x}=1.78$ can be calculated of the displacement series x=0,0.1,0.2,⋯,5.9,6 mm. Then, the weight coefficient $\mu_{i}$ can be expressed as:

(13) $$\mu \left(x\right)=0.22\cdot e^{-0.16{\left(x-3\right)}^{2}}.$$

After simplifying and calculating, the final decision model can be expressed as:

(14) $$g_{m}:u_{m}=sgn\left(\sum_{i=1}^{n}U_{i}\alpha_{i}K\left(u_{si},u_{sj}\right)+b\right),$$

where $K(u_{si},u_{sj})$ is the kernel function, $u_{m}$ is the output voltage, which is adjusted by the mid-scale adjustment function, and n=61 is the number of the samples (data with an interval of 0.1 mm are recorded once from 0 mm to 6 mm).

**Figure 7:**
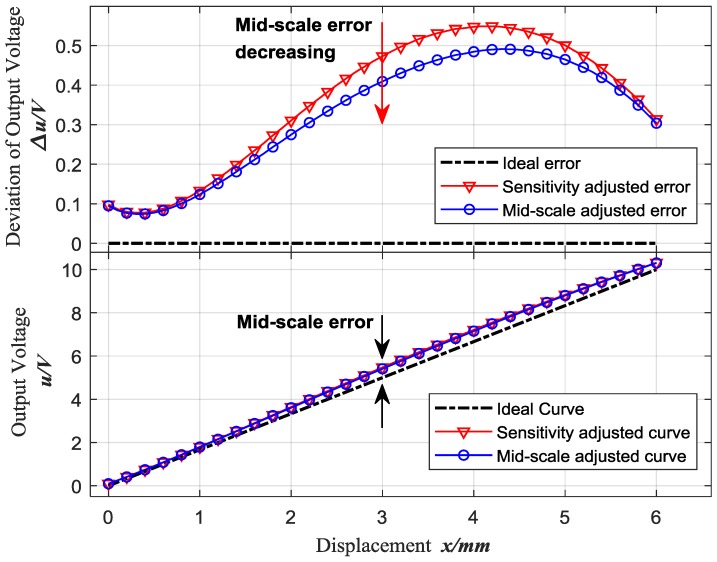
First result of mid-scale adjustment based on sensitivity adjusted curve for Sensor 1.

According to Figure 5, the mid-scale adjustment of ECDs is performed based on the sensitivity adjusted curve. Figure 7 shows the first result of mid-scale adjusted curve of sensor 1, and as well the output voltage deviation between the actual characteristic curve and the ideal characteristic curve, which is obviously reduced at the mid-scale.

### 3.3. Zero-Scale and Full-Scale Adjustment Function

After mid-scale adjustment, $u_{m}$ will be adjusted at the zero-scale, then at the full-scale, which are similar to the mid-scale adjustment process except the weight function.

In the process of zero-scale adjustment, the peak of adjustment amplitude is set at $\bar{x}=0$. Thus, the standard deviation $\sigma_{x}=5.33$ can be calculated of the displacement series x=0,0.1,0.2,⋯,5.9,6 mm in the case of half-Gaussian. Then, the weight coefficient $\mu_{i}$ can be expressed as:

(15) $$\mu \left(x\right)=0.07\cdot e^{-0.018x^{2}}.$$

The zero-scale adjustment function $g_{z}$ can be expressed as:

(16) $$g_{z}:u_{z}=sgn\left(\sum_{i=1}^{n}U_{i}\alpha_{i}K\left(u_{mi},u_{mj}\right)+b\right),$$

where $u_{z}$ is the output voltage, which is adjusted by the zero-scale adjustment function.

Then, in the process of full-scale adjustment, the peak of adjustment amplitude is set at $\bar{x}=6$. Thus, the standard deviation $\sigma_{x}=5.33$ can be calculated of the displacement series x=0,0.1,0.2,⋯,5.9,6 mm in the case of half-Gaussian. Then, the weight coefficient $\mu_{i}$ can be expressed as:

(17) $$\mu \left(x\right)=0.07\cdot e^{-0.018{\left(x-6\right)}^{2}}.$$

The full-scale adjustment function $g_{f}$ can be expressed as:

(18) $$g_{f}:u_{f}=sgn\left(\sum_{i=1}^{n}U_{i}\alpha_{i}K\left(u_{zi},u_{zj}\right)+b\right),$$

where $u_{f}$ is the output voltage that is adjusted by the full-scale adjustment function.

**Figure 8:**
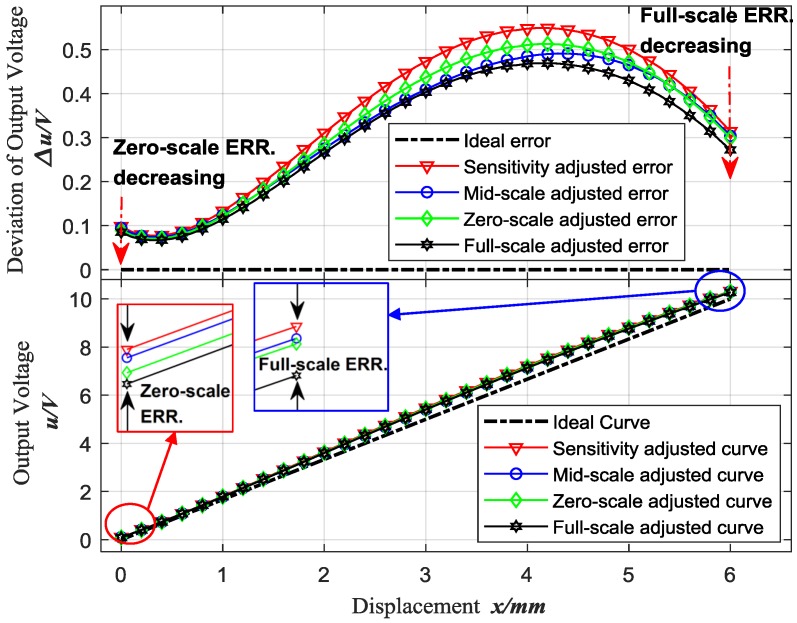
First results of zero-scale and full-scale adjustment based on mid-scale adjusted curve for Sensor 1.

Finally, Figure 8 shows the first loop results of the serial adjustments including sensitivity adjustment, mid-scale adjustment, zero-scale adjustment and full-scale adjustment based on the original curve of sensor 1, and as well the output voltage deviation between the actual characteristic curve and the ideal characteristic curve, which is obviously reduced at the mid-scale, zero-scale and full-scale, respectively.

### 3.4. Overall Adjustment Model

One-loop adjustment is completed as described above. Then, the one-loop adjustment model $c_{1}:u\to U$ can be expressed as $c_{1}=f_{z}\circ g_{m}\circ g_{z}\circ g_{f}$ and the residual is $\Delta =U-u\circ c_{1}$. The residual is optimized cyclically until Δ, and then the final adjustment function *L* of the output characteristic curve can be expressed as follows:

(19) $$\begin{array}{c} L=c_{1}\circ c_{2}\cdots c_{n},\\ s.t.\;\Delta =U-u\circ L\to 0, \end{array}$$

where $c_{i}\left(i=1,2,\cdots ,n\right)$ is the *i*th loop adjustment model.

**Figure 9:**
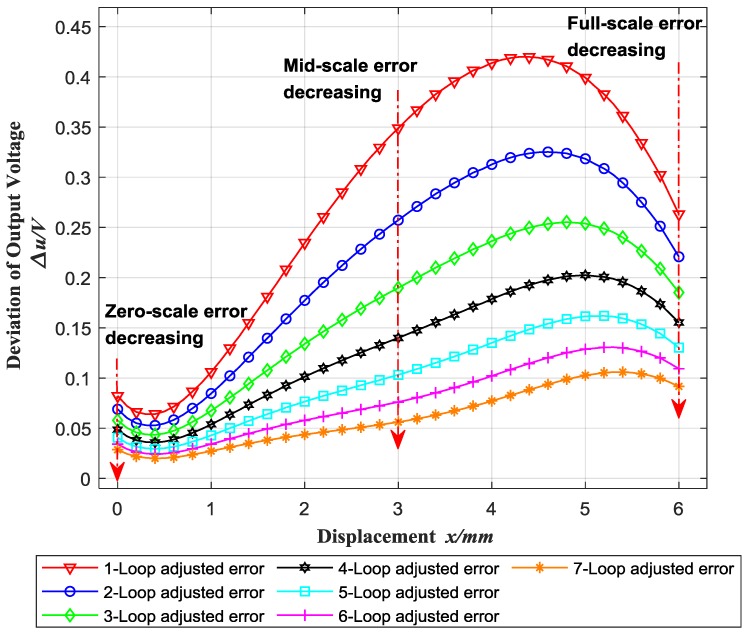
Overall adjustment results.

Consequently, the overall adjustment error results of Sensor 1 are shown in Figure 9. Through seven loops, the output voltage deviation is decreased from over 0.4 V to less than 0.1 V, which makes the sensitivity of Sensor 1 more than 99.9%, increasing 3.2% than the original linearity (96.77%). Table 3 shows the sensitivity and linearity of each sensor. The sensitivity after overall adjustment (about 7–15 loops) reaches 1.6644–1.6841 V/mm, and the linearity reaches 99.90–99.92%, which is increased by 1.9–5.0% over the original (95.13–98.00%).

## 4. Calibration Accuracy Verification

**Figure 10:**
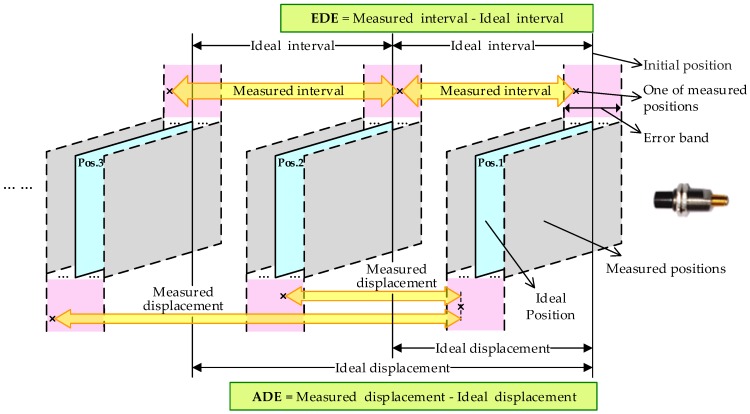
Verification schematic diagram.

In this paper, two methods are proposed to verify the adjusted calibration accuracy and precision: equidistance displacement error (EDE) and accumulated displacement error (ADE). The verification schematic diagram is shown in Figure 10.

### 4.1. Equidistance Displacement Error Verification

In the process of EDE verification, the ECDSs are placed off the measured target with a certain offset, which is defined as the initial position of the verification test. Then, the target is gradually moved away from the sensor, and the output voltages are recorded at intervals of 0.25 mm until reaching the full scale. Afterwards, the errors between the measured values of displacement interval and the true value (0.25 mm) are calculated. Table 4 shows the EDEs before and after adopting the adjusted calibration method proposed in this paper.

**Figure 11:**
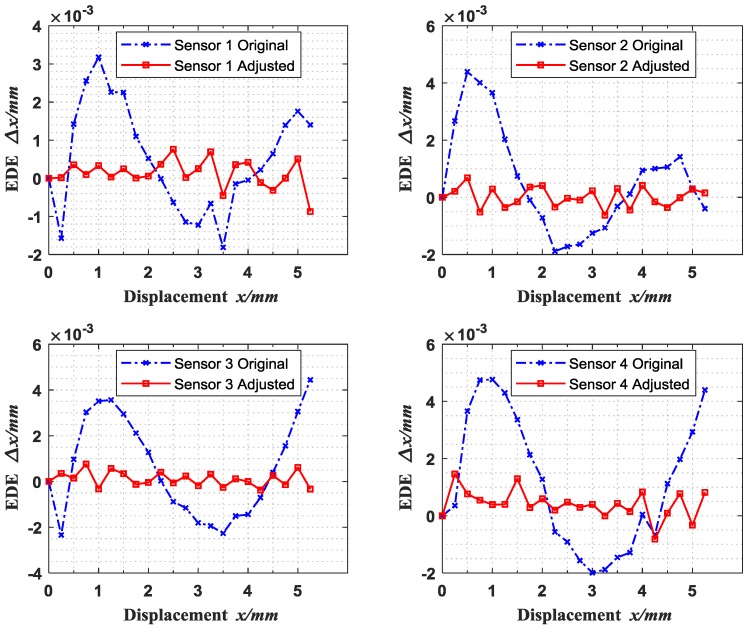
Equidistance displacement error (EDE) curves before and after adjusted.

Figure 11 shows the EDE curves before and after adjustment. From Table 4 and Figure 11, it can be seen that the average of EDE absolute values of Sensors 1 to 4 reduced from 1.18 μm, 1.43 μm, 1.86 μm and 2.06 μm to 0.29 μm, 0.29 μm, 0.27 μm and 0.52 μm, respectively, and the standard deviations are also reduced from 0.89 μm, 1.26 μm, 1.20 μm and 1.54 μm to 0.26 μm, 0.19 μm, 0.20 μm and 0.38 μm, which indicate that the accuracy of the equidistance displacement measurement is significantly improved.

### 4.2. Accumulated Displacement Error Verification

**Figure 12:**
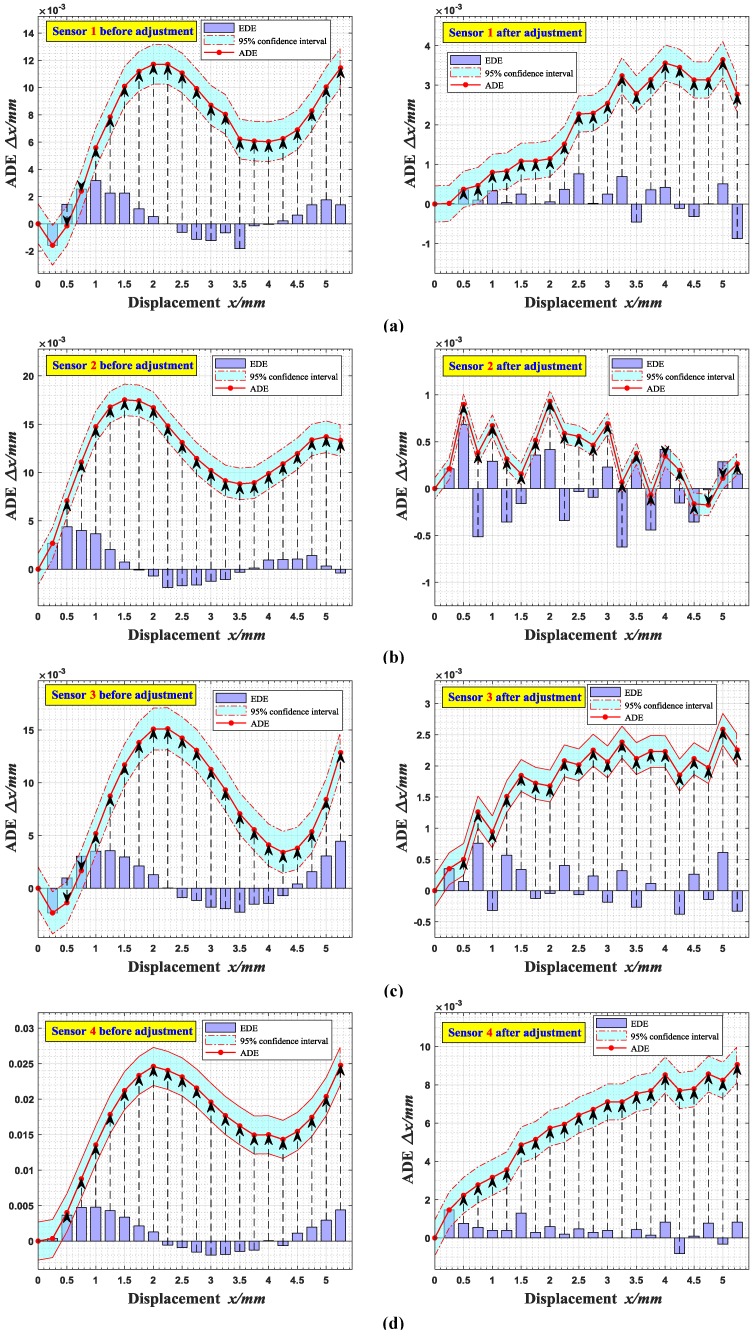
Accumulated displacement error (ADE) band curves of each sensor: (**a**) Sensor 1; (**b**) Sensor 2; (**c**) Sensor 3; (**d**) Sensor 4.

In the process of ADE verification, the ECDSs are also placed off the measured target with a certain offset, which is defined as the initial position. Then, the output voltages are recorded at intervals of 0.25 mm until reaching the full scale, and the errors between the measured displacement values and the true values are calculated. Figure 12 illustrates the accumulated displacement error band curves of each sensor, which is also significantly reduced after adjustment, from 11–25 μm to 1–10 μm, which indicates that the sensors have better accuracy.

**Figure 13:**
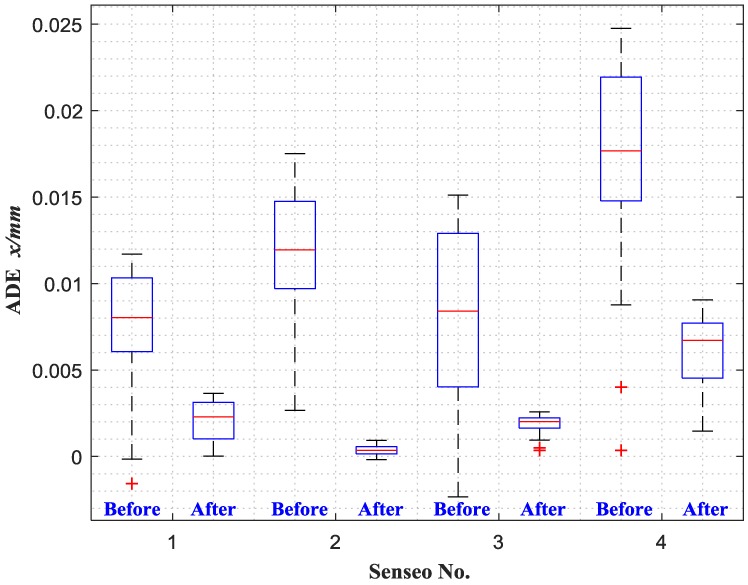
95% confidence interval of ADE for each sensor.

Then, the 95% confidence interval of accumulated displacement errors of each sensor are given in Figure 13, whose statistical data are shown in Table 5. In order to better reflect the measurement deviation characteristics of ECDSs, outliers are listed separately in the form of the red ‘+’. It can be seen that the original ADE medians of sensor 1 to 4 are 8.0314 μm, 11.9520 μm, 8.4100 μm and 17.6790 μm, respectively, which are reduced to 2.2914 μm, 0.3472 μm, 2.0165 μm and 6.7096 μm after adjustment. Moreover, the error fluctuation range of each sensor (from minimum to maximum) is also decreased from 13.2838 μm, 14.8441 μm, 17.4496 μm and 24.4002 μm (more than ±10 μm before adjustment) to 3.6297 μm, 1.1052 μm, 2.2354 μm and 7.5956 μm (less than ±5 μm after adjustment). It indicates that each adjusted sensor has a better accuracy, and as well the error fluctuation is markedly reduced. Therefore, the proposed calibration method can effectively improve both accuracy and precision of sensor calibration and measurement.

## 5. Conclusions

In this paper, a high-accuracy calibration method based on linearity adjustment is proposed for ECDSs. The sensitivity and linearity of the output curve of the sensor are adjusted based on the idea of partition, and, in addition, the μSVM method is employed to avoid the over-fitting problem. Results show that the linearity of the adjusted output characteristic curve is significantly increased by more than 1.9%, which reaches over 99.9%. In accuracy and precision experiments, EDE tests the deviation of the relative displacement between the measured value and the actual value, whose error range can reach 0.27–0.52 μm when the relative displacement is 0.25 mm for instance in this paper. In addition, ADE tests the deviation of the absolute displacement, which can reach 1–10 μm at the range of 6 mm. Then, the 95% confidence interval is calculated from repeated tests, and the repetition precision is less than ±5 μm. The improvement of the ECDSs’ performance by the proposed method provides a reliable guarantee for high accuracy displacement measurement in the quality control of the industrial process.

## Figures and Tables

**Table 1:** Original sensitivity and linearity of each sensor probe.

	Sensor 1	Sensor 2	Sensor 3	Sensor 4
**Output range (V)**	−0.9593–1.038	−0.9682–0.9760	−0.9906–0.9522	−1.0075–0.9813
**Sensitivity (V/mm)**	0.3329	0.3240	0.3238	0.3315
**Linearity**	96.77%	95.13%	98.00%	97.16%

**Table 2:** Sensitivity adjustment results.

	Sensor 1	Sensor 2	Sensor 3	Sensor 4
**Adjusted Output Range (V)**	0.098–10.3139	−0.103–9.7036	−0.1100–9.8800	−0.0852–10.3420
**Original Sensitivity (V/mm)**	0.3329	0.3240	0.3238	0.3315
**Adjusted Sensitivity (V/mm)**	1.7026	1.6344	1.6650	1.7379

**Table 3:** Results of adjusted sensitivity and linearity.

	Sensor 1	Sensor 2	Sensor 3	Sensor 4
**Number of Loops**	7	15	12	8
**Output Range (V)**	0.0286–10.0916	−0.0073–9.9788	−0.0133–9.9855	−0.0209–10.0837
**Sensitivity (V/mm)**	1.6772	1.6644	1.6665	1.6841
**Linearity (before adjustment)**	96.77%	95.13%	98.00%	97.16%
**Linearity (after adjustment)**	99.91%	99.90%	99.91%	99.92%
**Linearity increase**	3.2%	5.0%	1.9%	2.8%

**Table 4:** Equidistance displacement errors (EDEs) (μm) before and after adjustment (the value before ‘/’ represents the EDE before adjusted and the value after ‘/’ represents the EDE after adjusted).

	Minimum	Maximum	Average of EDE Absolute Value	Standard Deviation of EDE Absolute Value
**Sensor 1**	−1.81/−0.87	3.18/0.76	1.18/0.29	0.89/0.26
**Sensor 2**	−1.89/−0.62	4.39/0.69	1.43/0.29	1.26/0.19
**Sensor 3**	−2.33/−0.38	4.44/0.76	1.86/0.27	1.20/0.20
**Sensor 4**	−1.99/−0.82	4.77/1.46	2.06/0.52	1.54/0.38

**Table 5:** Accumulated displacement errors (ADEs) (μm) before and after adjustment.

	Minimum		Maximum		Median		Error Fluctuation Range	
	Before	After	Before	After	Before	After	Before	After
**Sensor 1**	−1.5738	0.0151	11.7100	3.6448	8.0314	2.2914	13.2838	3.6297
**Sensor 2**	2.6729	−0.1753	17.5170	0.9299	11.9520	0.3472	14.8441	1.1052
**Sensor 3**	−2.3386	0.3509	15.1110	2.5863	8.4100	2.0165	17.4496	2.2354
**Sensor 4**	0.3548	1.4598	24.7550	9.0554	17.6790	6.7096	24.4002	7.5956
